# Classification of In Vitro Phage–Host Population Growth Dynamics

**DOI:** 10.3390/microorganisms9122470

**Published:** 2021-11-30

**Authors:** Patricia E. Sørensen, Duncan Y. K. Ng, Luc Duchateau, Hanne Ingmer, An Garmyn, Patrick Butaye

**Affiliations:** 1 Department of Pathobiology, Pharmacology and Zoological Medicine, Ghent University, 9820 Merelbeke, Belgium; an.garmyn@ugent.be (A.G.); patrick.butaye@ugent.be (P.B.); 2 Department of Biomedical Sciences, Ross University School of Veterinary Medicine, Basseterre 42123, Saint Kitts and Nevis; 3 Department of Bacteria, Parasites and Fungi, Statens Serum Institut, 2300 Copenhagen, Denmark; duncan.ng@quadram.ac.uk; 4 Biometrics Research Center, Ghent University, 9820 Merelbeke, Belgium; luc.duchateau@ugent.be; 5 Department of Veterinary and Animal Sciences, University of Copenhagen, 1870 Frederiksberg, Denmark; hi@sund.ku.dk

**Keywords:** growth dynamics, phage–host interaction, phage therapy, bacteriophage, *Escherichia coli*

## Abstract

The therapeutic use of bacteriophages (phage therapy) represents a promising alternative to antibiotics to control bacterial pathogens. However, the understanding of the phage–bacterium interactions and population dynamics seems essential for successful phage therapy implementation. Here, we investigated the effect of three factors: phage species (18 lytic *E. coli*-infecting phages); bacterial strain (10 APEC strains); and multiplicity of infection (MOI) (MOI 10, 1, and 0.1) on the bacterial growth dynamics. All factors had a significant effect, but the phage appeared to be the most important. The results showed seven distinct growth patterns. The first pattern corresponded to the normal bacterial growth pattern in the absence of a phage. The second pattern was complete bacterial killing. The remaining patterns were in-between, characterised by delayed growth and/or variable killing of the bacterial cells. In conclusion, this study demonstrates that the phage–host dynamics is an important factor in the capacity of a phage to eliminate bacteria. The classified patterns show that this is an essential factor to consider when developing a phage therapy. This methodology can be used to rapidly screen for novel phage candidates for phage therapy. Accordingly, the most promising candidates were phages found in Group 2, characterised by growth dynamics with high bacterial killing.

## 1. Introduction

Bacteriophages (phages) are viruses that specifically infect bacteria. They are estimated to be the most abundant organisms on Earth (~10^31^ entities) and play a major role in shaping the microbial communities [1,2]. Phages are unable to replicate independently of a susceptible bacterial host, and their host range is determined by a combination of various factors, including host-binding protein specificity and bacterial phage-resistance mechanisms [3,4]. Bacteria can readily evolve resistance to phage infections though different mechanisms, which can result in distinct resistance phenotypes [5]. These can differ in whether the resistance is partial or complete, in the level of fitness cost associated with resistance, and in whether the mutation can be countered by the infecting phage. Consequently, these differences determine the effect of the phage infection on the bacterial population dynamics and the resulting community structure [6,7].

The therapeutic use of phages (phage therapy) represents an urgently needed alternative or supportive antibacterial agent to antibiotics to control bacterial pathogens [8,9,10,11]. However, the outcome of phage therapy is still difficult to predict [12]. Several factors can be involved, both animal host and bacterial host-related, as well as phage-related. Whether phages are able to infect and kill a susceptible target bacterial population at a specific site depends on the change in phage densities in different tissues of the host (pharmacokinetics) and the population dynamics of the phage–bacterial interaction (pharmacodynamics) [7]. Understanding the phage–bacterium interactions and population dynamics has been shown to be essential for more reliable in vitro and/or in vivo outcomes, and thereby essential for successful phage therapy development and application [7,13,14]. Several models have been developed to predict the behaviour and dynamics of phage-bacteria populations [15,16,17,18]. These models include parameters such as bacterial growth and mutation rate, phage adsorption rate, burst size, latent period, and virulence, as well as multiplicity of infection (MOI) [7,18]. While no single model to date has been able to capture all aspects of the complex phage–host interactions, together, suitable models can be selected to predict and explain basic behaviours of the population dynamics, as well as identify the dominant factors that contribute to the dynamics [7,15,17,18]. The dynamics of in vitro phage–host interactions may differ from those observed in vivo. This is similar to what is seen also with antibiotics [19,20]. Still, in vitro observations constitute a necessary initial step in understanding and predicting phage–host population dynamics in in vivo settings [7]. However, several models focus on the dynamics of only an individual phage and single host [17,18] and can fail to report what defines the dynamic(s). This study investigates the in vitro growth dynamics of a group of *Escherichia coli* (*E. coli*)-infecting phages (coliphages) and multidrug-resistant avian pathogenic *E. coli* (APEC). We define the specific patterns observed using a multiple parameter-based approach and estimate the parameter(s) (phage type, APEC strains, and MOI) that are key to each pattern. APEC (with O-serogroups O1, O2, or O78) was chosen as the bacterial model due to the significant problem it represents to poultry worldwide [21,22]. This pathogen causes a large range of extraintestinal infections, collectively referred to as colibacillosis, which are becoming harder to treat with the increasing resistance of APEC to different classes of antibiotics [23,24].

## 2. Materials and Methods

### 2.1. Bacterial Strains and Growth Conditions

The 10 avian pathogenic *E. coli* (APEC) strains are part of an in-house collection that were isolated from clinical poultry faeces samples suspected of APEC infection. Samples were collected in Belgium during 2013 to 2014 by the Animal Health Care Flanders (Torhout, Belgium). Strains were grown in Luria Bertani (LB) broth (Miller) (Sigma-Aldrich, Saint Louis, MO, USA) or on LB agar supplemented with 1.5% bacteriological agar no. 1 (*w*/*v*) (Oxoid, Basingstoke, UK) overnight (16–18 h) at 37 °C unless stated otherwise. Broth cultures were incubated with shaking (120 rpm). Strains were stored at −80 °C in LB broth supplemented with 15% glycerol (Sigma-Aldrich, Saint Louis, MO, USA).

### 2.2. Genomic DNA Extraction and Sequencing

Bacterial genomic DNA was extracted using Qiagen’s DNeasy Blood and Tissue Kit (Qiagen, Hilden, Germany), with the subsequent library construction using the Nextera XT Kit (Illumina, Little Chesterford, UK), and sequenced using a 300-cycle kit on the Illumina NextSeq platform according to the manufacturer’s instructions.

### 2.3. Bacterial Genome Analysis

The bifrost platform (https://github.com/ssi-dk/bifrost (accessed on 15 January 2021)), v1.1.0 was used for quality control validation of the raw reads data. The raw reads were de novo assembled using SPAdes v3.12.1 [25] and MLST typed with the MLST command line tool (https://github.com/tseemann/mlst (accessed on 15 January 2021)), v2.19. The serotypes were predicted using SerotypeFinder, v2.0 [26]. ABRicate v1.0.1 (https://github.com/tseemann/abricate (accessed on 18 June 2021)) with default options was used to screen the assembled contigs for antimicrobial resistance genes with ResFinder [27] and the Comprehensive Antibiotic Resistance Database (CARD) [28]. Virulence genes were identified using ABRicate with data from the Ecoli_VF database. Prophage regions were identified using the PHAge Search Tool Enhanced Release (PHASTER) tool [29]. The Rapid Annotation using Subsystem Technology (RAST) server and the SEED viewer, version 2.0 [30] were used for identification of the coding sequences (CDSs) and initial annotation of the APEC genomes.

### 2.4. Bacteriophage Isolation, Purification, and Enumeration

A total of 18 lytic coliphages were used in this study. These were selected from the collection based on their genomic diversity. Phages were isolated from poultry faecal material using *E. coli* laboratory strain K514, sequenced, and identified as described before [31]. Phage stocks were stored at titres of 1.2 × 10^7^ to 4.5 × 10^10^ plaque-forming units (PFU)/mL at 4 °C. Working stocks used for phage infectivity and phage–host growth experiments were kept at titres of ~10^10^ PFU/mL.

### 2.5. Phage Infectivity and Phage–Host Growth Dynamics

Bacterial overnight cultures were used, and the cell concentration was adjusted to ~10^8^ colony-forming units (CFU)/mL for every experiment. Bacterial solutions were inoculated with 20-µL phages yielding initial MOIs of 10, 1, or 0.1. All bacterial reduction curves were generated using 96-well plates with working volumes of 200 µL. Experiments were performed in triplicate. For the experiments of the susceptible combinations, the experiment was performed on a duplicate plate at another time. A well of phage-free bacterial culture and a well of bacteria-free phage culture were included on every plate as control experiments in addition to one media blank for reference. The optical density (OD) was measured for a wavelength of 600 nm (OD600) with the Thermo Fisher Scientific Multiskan GO Microplate Spectrophotometer, v1.01.12, and the data were recorded using SkanIt software, v6.0.2.3. The OD600 was measured with fast measurement mode and no pathlength correction or use of transmittance, and the measurements were taken immediately after inoculation and then at regular intervals of 30 min afterward for 22 h. The incubation temperature was 37 °C, and shaking was continuous at a medium speed.

Growth curves were obtained by plotting OD600 values after baseline adjustment against time. Phage infectivity was defined based on endpoint measurements. Successful phage infection was defined as OD600: <0.2, somewhat successful infection was defined as OD600: 0.2–0.5, and failed phage infection as OD600: >0.5. The phage–host growth dynamics were assessed based on measurements throughout the experiment.

### 2.6. Assessing the Effect of Phage Species, APEC Strain, and MOI

The two different response variables, PhageScore [32] and local virulence score [16], were derived to assess the effect of the phage species, APEC strain, and MOI on the growth dynamics. For the PhageScore method, the area under the growth curve (AUC) was determined for the complete study period for each treatment combination and for the corresponding control, i.e., without phage, and the ratio of the difference between the control and the treatment over the control (multiplied with 100) was calculated. For the local virulence score, the same was done, but only measurements until the stationary phase in the control group were taken into consideration, i.e., until the timepoint before the maximum OD value.

A fixed effects model with a normally distributed error term was used, and the phage, bacterium, and MOI, as well as all two-way interactions, were included in the model. F-tests were performed at the 5% significance level to assess the effects of the different factors. Finally, the Pearson correlation coefficient between the PhageScore and the virulence score was calculated.

### 2.7. Classification of Phage–Bacterium Growth Dynamics

To classify the growth dynamics of phage–host interactions based on the OD measurements, we applied two statistical data mining techniques: nonmetric multidimensional scaling (NMDS) ordination and principal component analysis (PCA), with hierarchical agglomerative clustering. A NMDS ordination plot (Euclidean distance) using the vegan package in R (http://www.R-project.org/ (accessed on 18 April 2021)) [33] was applied to quantify and visualise the pairwise dissimilarity between samples of each timepoint. The stress value was determined to access how well the data were transformed. A stress value between 0.02 and 0.01 was considered an acceptable fit, and <0.01 was considered a good fit. Metadata was included using the envfit function to determine the effect of each factor (phage species, bacterial strain, and MOI). Using the factoextra package the right number of groups (of the growth dynamics pattern) was determined using bootstrap values = 100 and hierarchical agglomerative clustering (“ward.D” method). The results were visualised using the ggplot2 package. A PCA was performed as verification (sanity check) of how much of the variability in the data is explained by each factor and how much of the total variability is captured. Subsequent subclustering of the designated groups was performed as described above for the complete dataset.

### 2.8. Repeatability of Group Assignments

The dplyr package in R was used to determine if replicates of the same phage–bacterium–MOI combination were placed in the same group and subgroup.

## 3. Results

### 3.1. APEC Strains

Whole-genome sequencing (WGS) of the bacterial genomes yielded a total of 2,673,858–4,825,608 paired-end reads for each of the 10 isolates, with an average coverage of 77–142-fold. The characteristics based on the WGS analysis are summarised in Table 1. The strains belonged to one of four serotypes and MLST sequence types (ST): Stains B1, B4, and B8 had serotype O1:H7 (ST95); stains B5 and B10 had serotype O2:H5 (ST355); strains B2 and B3 had serotype O78:H4 (ST117); and strains B6, B7, and B9 had O78:H9 (ST23). Each bacterial genome comprised between 5 and 13 prophage regions, including a total of 40 different prophages (Supplementary Table S1).

### 3.2. Coliphages

The coliphages used in this study were previously characterised [31]. The characteristics are summarised in Table 2. The phages belonged to one of seven different genera. *Tequatrovirus* phages included P1, P2, and P9. *Mosigvirus* phages included P3, P5, and P6. *Guelphvirus* phages included P4, P13, and P14. *Hanrivervirus* phages included P7, P8, and P16. *Felixounavirus* phages included P10, P12, P17, and P18. *Tequintavirus* phages included P11. *Warwickvirus* phages included P15.

### 3.3. Phage Infectivity

**Figure 1:**
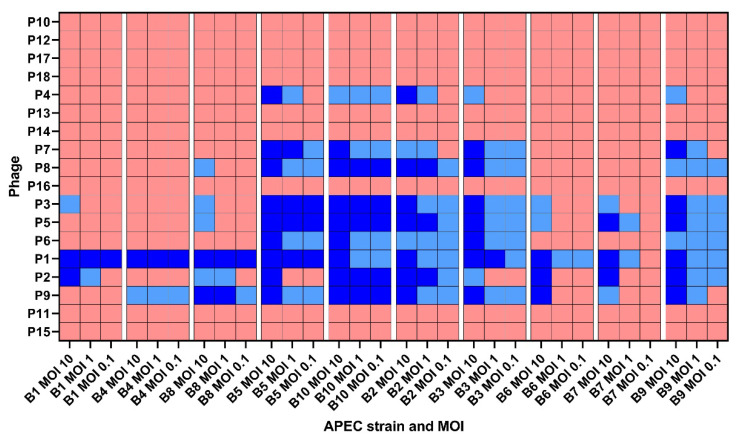
Infectivity of the tailed coliphages based on the endpoint optical density (OD). P = phage. B = bacterium. MOI = multiplicity of infection. Ratio = phage:APEC. OD was measured at 600 nm (OD600). Dark blue = final OD600: <0.2, light blue = final OD600: 0.2–0.5, and red = final OD600: >0.5. The final OD was determined based on the average of thirty-nine replicates.

The infectivity of the 18 coliphages against each of the 10 APEC strains at MOI 10, 1, and 0.1 is shown in Figure 1. The levels of infectivity of the tested coliphages were highly variable, varying between 0% and 100% of the tested APEC strains. Variations in the degree of infection (successful, somewhat successful, or failed) were detected, with the most successful infections at MOI 10 and least at MOI 0.1. Phage P1 was shown to be the most infective, as it was the only one able to infect all APEC strains at all MOI tested, except for B7-MOI 0.1. Phages P2, P3, and P9 were able to infect nine of the APEC strains, excluding B4, B4, and B1, respectively. Phage P5 was able to infect eight of the APEC strains, excluding B1 and B4. Phage P8 was able to infect six of the APEC strains, not including B1, B4, B6, and B7. Phages P4, P6, and P7 were able to infect five of the APEC strains, excluding B1, B4, B6, B7, and B8. The nine phages P10–18 were not able to infect any of the 10 APEC strains.

### 3.4. Assessing the Effect of Phage Species, APEC Strain, and MOI

**Figure 2:**
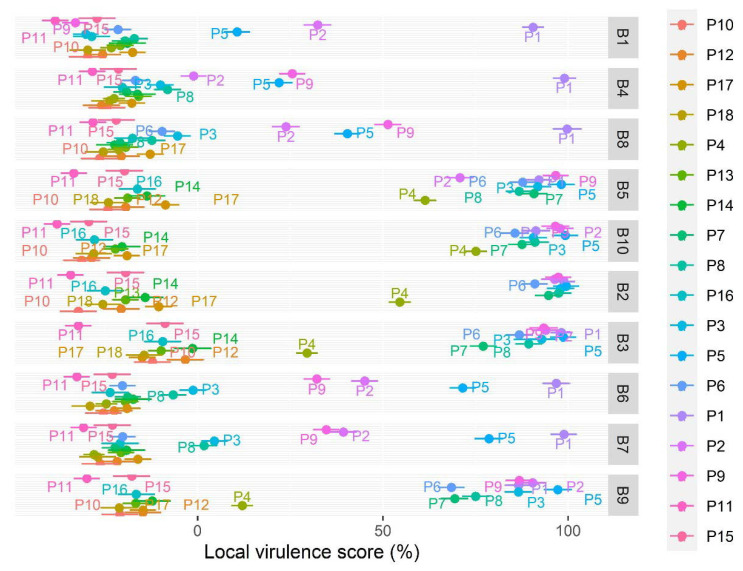
Virulence score average by bacteria and phage. A higher virulence score correlates with a higher virulence of the phage and higher bacterial growth reductions. The standard error is shown. A total of 2869 phage–host combination experiments were included.

A total of 2869 phage–host combination experiments were performed. The average local virulence score and its standard error for each phage–bacterium combination are shown in Figure 2. All factors were significant, i.e., MOI (F_2,2495_ = 938.8, *p* < 0.0001), phage (F_17,2495_ = 1095.8, *p* < 0.0001), and bacterium (F_9,2495_ = 459.5, *p* < 0.0001), and the interactions too but to a lesser extent, i.e., phage–bacterium (F_153,2495_ = 42.8, *p* < 0.0001), phage–MOI (F_34,2495_ = 10.7, *p* < 0.0001), and bacterium–MOI (F_18,2495_ = 9.7, *p* < 0.0001). The phage effect was thus more pronounced than the bacterium effect. Based on the virulence scores, the greatest reduction in bacterial growth of the six strains B1, B2, B4, B6, B7, and B8 was observed with phage P1 and with phage P5 for the four strains B3, B5, B9, and B10.

**Figure 3:**
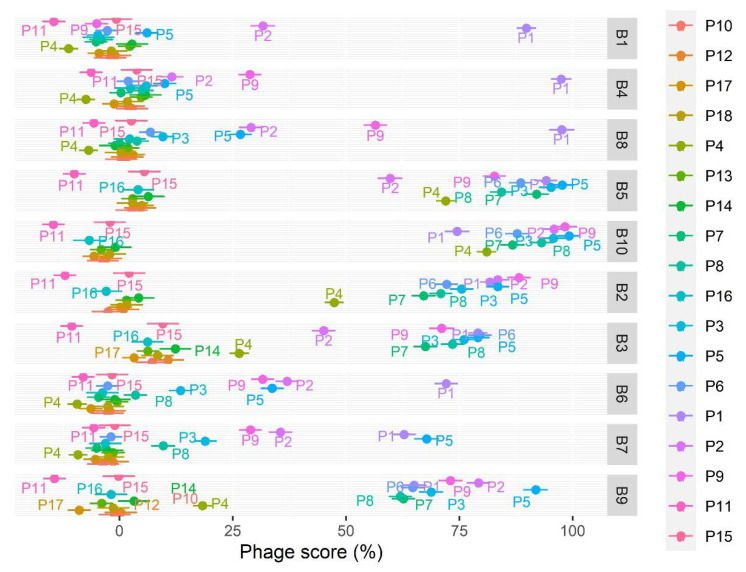
PhageScore average by bacteria and phage. A higher PhageScore correlates with a higher efficiency of the phage and higher bacterial growth reduction. A total of 2869 phage–host combination experiments were included. The standard error is shown.

The average PhageScore and its standard error for each phage–bacterium combination are shown in Figure 3. All factors were significant, i.e., MOI (F_2,2495_ = 447.9, *p* < 0.0001), phage (F_17,2495_ = 693.6, *p* < 0.0001), and bacteria (F_9,2495_ = 364.6, *p* < 0.0001), and the interactions too but to a lesser extent, i.e., phage–bacteria (F_153,2495_ = 38.1, *p* < 0.0001), phage–MOI (F_34,2495_ = 13.0, *p* < 0.0001), and bacteria–MOI (F_18,2495_ = 12.5, *p* < 0.0001). The phage effect was thus also more pronounced than the bacterium effect when looking at the PhageScore. Based on the PhageScore, the greatest reduction in bacterial growth of the four strains B1, B4, B6, and B8 was observed with phage P1 and with phage P5 for the four strains B5, B7, B9, and B10. The greatest reduction of B2 and B3 growth was observed for phages P9 and P6, respectively.

The Pearson’s correlation coefficient between the local virulence score and the PhageScore was equal to 0.94.

### 3.5. Classification of Phage–Bacterium Growth Dynamics

**Figure 4:**
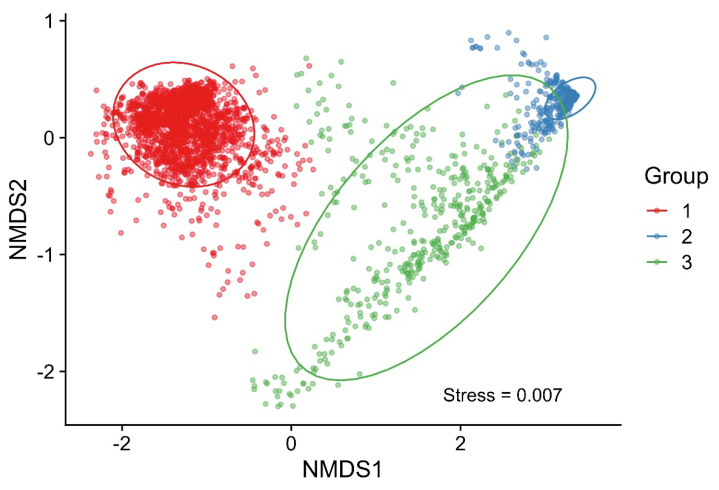
Groupings based on a nonmetric multidimensional scaling (NMDS) analysis. The analysis resulted in three groups. Group 1 comprises resistant combinations with bacterial growth, as well as phage-free controls. Group 2 comprises susceptible combinations with bacterial killing. Group 3 comprises in-between combination. A total of 2869 entities are shown on the plot. Ellipses indicate a 95% confidence level based on a multivariate t-distribution. Stress < 0.01 = a good fit.

The phage–host growth dynamics were classified based on the OD measurements from a total of 2869 phage–host combination experiments. The NMDS analysis grouped the data into three different groups (Figure 4). Group 1 comprised resistant phage–host combinations characterised by bacterial growth, Group 2 comprised fully susceptible combinations characterised by bacterial killing, and Group 3 comprised in-between combinations. A two-dimensional plot was considered appropriate, as the generated stress value was below 0.01 (0.007). Growth dynamics curves associated with each combination experiment and phage-free control culture are shown in Supplementary Figure S1. The principal component analysis (PCA) captured a total of 97% of the variance and showed similar groupings (Supplementary Figure S2).

**Figure 5:**
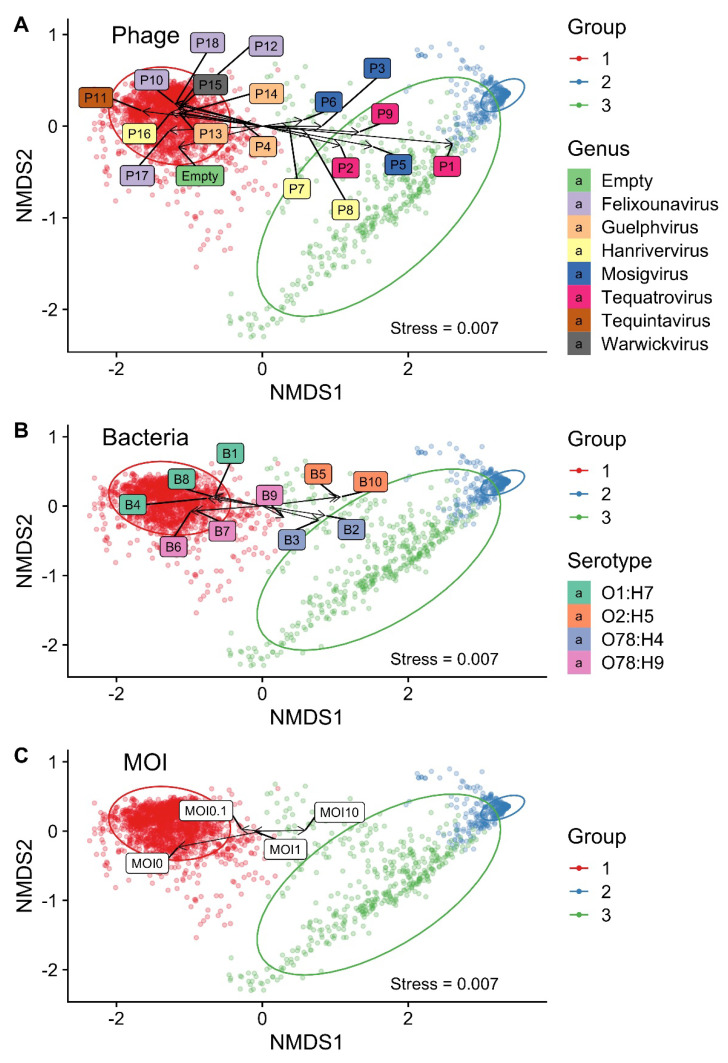
A nonmetric multidimensional scaling (NMDS) plot of the factors driving the grouping. (**A**) Effect of the phage type, including phage-free controls (empty). (**B**) Effect of the bacterial strain. (**C**) Effect of the multiplicity of infection (MOI). MOI 0 represents phage-free controls. A total of 2869 entities were divided into three groups. Group 1 represents resistant combinations/bacterial growth. Group 2 represents susceptible combinations/bacterial killing. Group 3 represents in-between combinations. Arrows indicate the strength and direction of the correlation. Ellipses indicate a 95% confidence level based on a multivariate t-distribution. Stress < 0.01 = a good fit.

NMDS on a two-dimensional graph was applied to investigate how the factors (bacterial strain, phage species, and MOI) affect the groupings (Figure 5). The 10 phages P4 and P10–18 were associated with bacterial growth (Group 1), of which P11 had the greatest association (similar to the phage-free controls), followed by P17, P4, P16, P13, P18, P10, P15, P12, and P14 (Figure 5A). Accordingly, the greatest bacterial growth was associated with the *Demerecviridae* phage belonging to the *Tequintavirus* genus. *Myoviridae* phages belonging to the *Felixounavirus* genus and *Drexlerviridae* phages belonging to the *Guelphvirus* or *Warwickvirus* genera were also associated with bacterial growth. Phages associated with more bacterial killing (Groups 2 and 3) included the eight phages P1–3 and P5–9, of which P1 had the strongest association, followed by P5, P9, P2, P3, P8, P6, and P7. A few P4 combinations were also found in Group 3. Accordingly, the greatest bacterial killing was associated with *Myoviridae* phages belonging to the *Tequatrovirus* genus. *Myoviridae* phages belonging to the *Mosigvirus* genus were also associated with bacterial killing, as well as two out of three *Hanrivervirus* phages (*Drexlerviridae* family). The five bacterial strains B1, B4, B6, B7, and B8 were associated with bacterial growth (Group 1). This included all three O1 serotype strains (B1, B4, and B8), as well as two O78 serotype strains (Figure 5B). The other five strains: B2, B3, B5, B9, and B10, were associated with more bacterial killing and included two O2 serotype strains (B5 and B10) and three O78 serotype strains. The overall effect of the bacterial factor on the plot was less compared to the effect of the phage factor. A high MOI (MOI 10) was associated with more bacterial killing, and a low MOI (MOI 0.1) was associated with less killing (Figure 5C).

**Figure 6:**
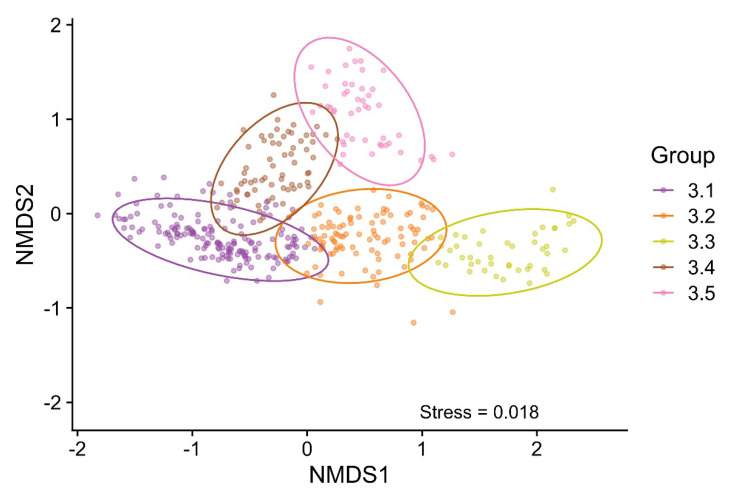
Subgrouping of Group 3 based on nonmetric multidimensional scaling (NMDS). Group 3 (*n* = 419) was divided into five subgroups. Subgroups 3.1 (*n* = 167), 3.2 (*n* = 95), and 3.3 (*n* = 43) represent combinations with the initial bacterial killing followed by exponential bacterial growth and the stationary phase. Subgroups 3.4 (*n* = 66) and 3.5 (*n* = 48) represent combinations with the initial bacterial growth followed by the stationary phase. Ellipses indicate a 95% confidence level based on a multivariate t-distribution. Stress < 0.02 = an acceptable fit.

Based on the NMDS analysis, Group 3, including 419 entries, was divided into five subgroups (Figure 6). Only combinations with phages P1–P9 were found. The growth curves associated with each subgroup are shown in Supplementary Figure S3.

**Figure 7:**
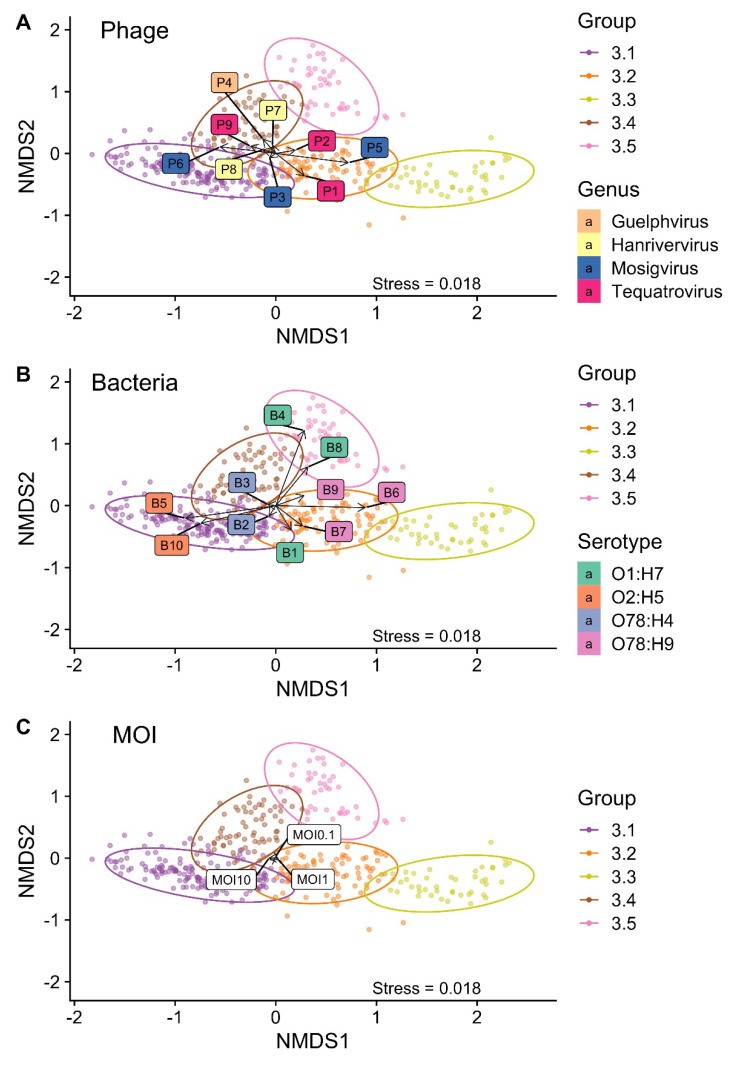
A nonmetric multidimensional scaling (NMDS) plot of the factors driving the Group 3 subgrouping. (**A**) Effect of the phage type. (**B**) Effect of the bacterial strain. (**C**) Effect of the multiplicity of infection (MOI). Arrows indicate the strength and direction of the correlation. Ellipses indicate a 95% confidence level based on a multivariate t-distribution. Stress < 0.02 = an okay fit.

NMDS on a two-dimensional graph was applied to investigate how the factors (bacterial strain, phage species, and MOI) affect the Group 3 groupings (Figure 7). The association of phage P3 was in the direction of subgroup 3.1; however, the association was very weak. Phages P1 and P5 had a strong association with subgroup 3.2, while P2 and P3 had a weak association. A P5 association was in the direction towards subgroup 3.3. The remaining five phages, including P4, P6, P7, P8, and P8, were associated with subgroup 3.4. P6 was the only phage with a strong association (Figure 7A).

Bacterial strains B5 and B10 (O2 serotype) had a strong association with subgroup 3.1, and B2 (O78) had a weak association. B1 (O1), as well as O78 strains B6, B7, and B9, had an association with subgroup 3.2. B6 was the only stain with a strong association, as well as the only strain driving the plot towards subgroup 3.3. No strains were driving the plot towards subgroup 3.4. Strain B3 was found in this subgroup but showed a very weak/no association. O1 strains B4 and B8 had strong associations with subgroup 3.5 (Figure 7B). The MOI did not have a strong association with any of the subgroups (Figure 7C).

### 3.6. Description of Defined Growth Dynamics Patterns

**Figure 8:**
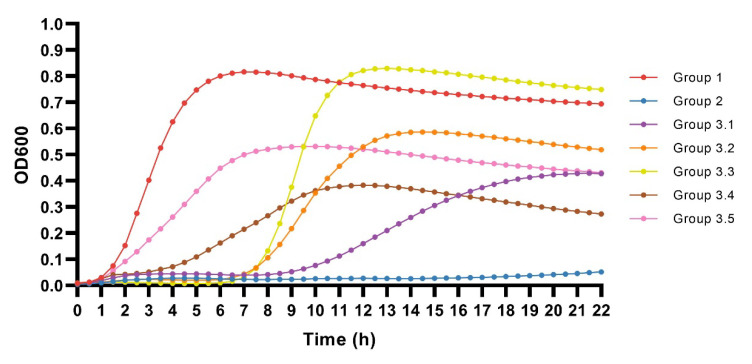
Growth dynamic patterns of the coliphage–APEC coculture combinations. Patterns are defined based on the average optical density (OD) value for each timepoint for each group.

Based on the bacterial growth (OD) detected in the 2869 phage–host combination experiments, three different growth dynamics patterns groups, including five subgroups, were defined (Figure 8). The groups included: (1) combinations with a fully resistant bacterial growth pattern; (2) phage–host combinations with a fully susceptible pattern, showing minimal or no bacterial growth; and (3) combinations with one of five in-between patterns characterised by delayed growth, lower killing, or variable killing of the bacterial cells. For all dynamics patterns except Group 1, the phage effect on the bacterial growth kinetics was observed within only a few hours of incubation. When the stationary phase was reached, the bacterial density remained stable throughout all the coculturing experiments.

Group 1 (bacterial growth): The bacterial growth continued to increase during the first 7 h of incubation until the cultures reached the stationary phase. The final OD600 was ~0.7. The pattern observed showed logistic growth as under standard conditions without phage present and could be explained by the presence of naturally phage-resistant strains, where the phage is unable to infect and has no effect on the growth. Group 2 (high level of bacterial killing): The bacteria were lysed and never recovered. A single small peak of bacterial growth followed by bacterial killing was observed in some cases but was not reflected in the average OD growth curve. Group 3.1 (initial bacterial killing followed by bacterial growth): Prolongation of the lag phase with no or low bacterial growth for ~9 h was observed, followed by a slow increase in bacterial growth until the stationary phase was reached. The final OD600 was ~0.45. The greatest variations were seen in this subgroup (Supplementary Figure S3). Group 3.2 (initial bacterial killing followed by bacterial growth): Prolongation of the lag phase with no or low bacterial growth was observed, followed by a sharp increase in the bacterial density after ~7 h of incubation before reaching the stationary phase. The final OD600 was ~0.5. Group 3.3 was characterised in a similar way as Group 3.2, except for a higher final OD600 of ~7.5, the highest observed for the seven different patterns. Group 3.4 (impaired bacterial growth): Increase of the bacterial growth was observed during the first ~9.5 h of incubation until the cultures reached the stationary phase. Impaired growth was observed compared to the Group 1 and Group 3.5 patterns, with a final OD600 of ~0.26. Group 3.5 was similarly characterised by impaired bacterial growth. Increase of the bacterial growth was observed during the first ~12 h of incubation until the cultures reached the stationary phase. Impaired growth was observed compared to the Group 1 patterns, with a final OD600 of ~0.45 (similarly to Group 3.1).

### 3.7. Repeatability of Group Assignments

**Figure 9:**
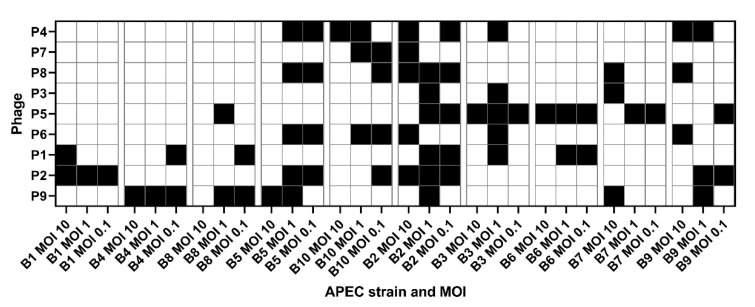
Overview of the discrepancies of the groupings of the phage–APEC–MOI combination replicates. P = phage. B = bacterium. MOI = multiplicity of infection. White = no discrepancies. Black = one or more discrepancies. Sixty-nine technical replicates were included for each combination. Combinations with P10–18 were not included, as no discrepancies were detected.

All replicates, including the phage–bacteria–MOI combinations with phage P10–P18, were found in same NMDS group (Group 1). Some discrepancies in the group assignments were seen for replicates of specific combinations, including eight phage P1 combinations, 11 P2 combinations, three P3 combinations, nine P4 combinations, 12 P5 combinations, seven P6 combinations, three P7 combinations, seven P8 combinations, and 10 P9 combinations (Figure 9). Most discrepancies included combinations with replicates grouped in Group 2 (bacterial killing) and Group 3.1 (in-between and bacterial killing), replicates grouped in Group 2 and Group 3.2/3.3 (initial bacterial killing followed by bacterial growth), or replicates grouped in Group 1 (bacterial growth) and Group 3.4/3.5 (in between dynamics and bacterial impaired growth) (Supplementary Tables S2 and S3).

## 4. Discussion

It has become clear that successful phage therapy development and application, among others, depend on an understanding of the phage–host interactions and population dynamics [34]. This study presents the classification of bacterial growth dynamics in the presence of lytic phages using two statistical data mining techniques: NMDS and PCA. OD as the input is a fast and data-rich screening method for in vitro phage–host growth dynamics [35]. This approach captures the ongoing dynamics and produces a quantitative high-throughput data to determine the phage–host range, phage virulence/infectivity, and bacterial phage resistance development [16,32,36]. These parameters can be important as pharmacodynamic (PD) parameters and also include part of the pharmacokinetics (PK), as it assesses the potential increase of the treatment dose. This would not be the case when relying on a single endpoint measurement. However, it is understood that ODs do not differentiate between viable and dead cells, and as such, there was no exact link between the OD values and bacteria viability but, nevertheless, a good proxy. The repeatability of the NMDS grouping was shown to be acceptable. Fully natural phage-resistant combinations were clearly identified. Most discrepancies observed between groupings of the replicates from the same phage–APEC–MOI combinations can be explained by grouping cut-off values. Replicates grouped in Group 1 and Group 3.4/3.5 included dynamics characterised by higher or lower bacterial growths. Replicates grouped in Group 2 and Group 3.1 included dynamics characterised by the initial bacterial killing, with no or low subsequent growth.

The optimal number of clusters/groups varied depending on the method used. In this study, we chose five clusters. However, subgroups 3.2 and 3.3 and subgroups 3.4 and 3.5 were relatively similar and could potentially be combined, resulting in only three Group 3 subclusters, and this would also create fewer discrepancies in the group allocations. The discrepancies due to biological variations can be expected due to the spontaneous emergence of phage-resistant variants after varying the incubation time (Group 2 vs. Groups 3.1, 3.2, and 3.3). It is also possible for a very small (partially) resistant sub-population of bacteria to be naturally present in the culture at the start of the experiment [17].

Various factors affecting the phage PK/PD have been described using mathematical and experimental models [15,17,18,37,38]. In this study, the influence of the factors (phage type, bacterial strain, and MOI) on the observed growth patterns was determined. Previous studies have highlighted the MOI influence on phage therapy, and recently, a fast microtiter plate assay for determination of the optimum MOI for a coliphage was further described [39]. However, in this study, we found the MOI to have a less significant effect on the phage–host growth dynamics outcome compared to the phage species. Furthermore, a recent study suggested that the description of MOI alone is not sufficient, as the concentration, particular to the bacteria, can significantly affect the results [40]. Therefore, in this study, the MOI at all the tested values was based on a fixed bacterial concentration.

A quantitative assessment of the phage lytic activity using the virulence score and PhageScore across a large dataset allowed direct comparisons of individual phages. In contrast to a single OD endpoint measurement and the well-established plaque assay, these methods (virulence score and PhageScore) captured the dynamics of phage infection, including bacterial (re)growth after prolonged growth inhibition or lysis. Additionally, compared to the overlay-based efficiency of plating assays and direct spot testing, these methods represent an accurate and less cumbersome and time-consuming approach and do not depend on the subjectivity and/or experience of the observer [41]. However, upscaling of this approach depends on the availability of a high-throughput plate reader. In accordance with previous findings, we found the two methods highly comparable, showing similar properties/values for the studied phages [32,42]. In this study, whenever the bacteria were able to grow and reach the stationary phase, the growth remained stable throughout all the experiments. If a second peak appeared or the growth started to decrease (after reaching the stationary phase), the comparability of the local virulence score and the PhageScore would be reduced. In this study, we only analysed the growth dynamics of cocultures of a single phage type and bacterial strain. However, both the virulence score and PhageScore have previously been used to compare phage combinations for use in phage therapy cocktails [32,41]. In future studies, the inclusion of mixed phage cultures (phage cocktails), preferably targeting different host receptors, may provide further indications of their potential as therapeutics against pathogenic target bacteria [17]. Accordingly, for future applications, the inclusion of phage, as well as bacterial, traits may be required for classification.

Given their great abundance and diversity, multiple candidate phages might be available to infect a target host; yet, we still lack a better understanding of which phage would perform best [43]. One approach to identify the cause(s) of treatment success is to compare the characteristics of phages with high success rates with those of phages with low success rates. The characteristics differing between these two groups of phages become candidates for causation. In this study, *Tequintavirus* phages (associated with bacterial growth) and *Tequatrovirus* phages (associated with bacterial killing) would be great candidates for comparisons. Accordingly, the inclusion of phage characteristics, such as the phage receptor, adsorption rate, latency period, burst size, and virion size, may provide further explanation of the phage–host dynamics and may help predict the phage therapy efficacy [44,45,46].

Phage therapy is, by its nature, a strongly selective treatment [17]. Accordingly, when selecting phages for therapeutical application, the emergence of phage-resistant bacteria should be taken into consideration [7]. Bacteria can develop resistance against phages through various mechanisms, including the modification of phage receptor-encoding genes, innate immune systems (such as CRISPR-Cas), and the presence of prophages in the bacterial genome [47,48]. Here, O1 serotype strains B1, B4, and B10 were associated with natural phage resistance/high levels of bacterial growth, whereas the strains with serotype O2 and serogroup O78:H4 were associated with phage susceptibility/bacterial killing. Moreover, all phage–host combinations including P10–18 were found in Group 1 (fully resistant combinations). These phages would be excluded as candidates for phage therapy targeting the selected APEC strains. P1 was the only phage not included in any Group 1 combinations and showed the greatest bacterial killing potential. Accordingly, phages only included in Group 2, characterised by high bacterial killing, are considered the most promising candidates for phage therapy. Multiple phage–host–MOI combinations were characterised by initial bacterial killing followed by bacterial growth (subgroups 3.1, 3.2, and 3.3), suggesting the emergence of phage-resistant bacterial variants. Whether phages found in these subgroups should be considered suitable for phage therapy depends on their specific applications, and further studies are needed to determine if the initial inhibition of bacterial growth for ~7 h is sufficient to clear out the infection.

Although in vitro experiments do not capture many in vivo realities, such experiments can give significant insights into the phage–host dynamics and lead to interesting predictions, which could be useful in phage therapy and exploited in appropriately designed in vivo models [13]. Recently, a framework (Clinical Phage Microbiology) with recommendations for in vitro identification and the evaluation of phages intended for treatment was published [42]. One step of the framework pipeline included determination of the growth kinetics of liquid cultures and highlighted the need for a standardised quantitative assessment with reproducible scoring. The methodology applied here constituted such an assessment and may help to improve the standardisation of the quantitative evaluation of the phage candidates.

## 5. Conclusions

Our methodology assessing the host–phage interaction in vitro provided a high-throughput method for classifying the bacterial growth dynamics in the presence of virulent coliphages using measurements of bacterial growth by OD as the inputs. The established in vitro model was not only used to gain a better understanding of the phage PK/PD but can also be applied as a screening method for selecting new suitable phage candidates for therapeutic applications against pathogenic target bacteria. However, to fully understand the complexity of these phage–host dynamics, the underlying mechanisms behind these different interactions need to be deciphered.

## Figures and Tables

**Table 1:** Avian pathogenic *Escherichia coli* (APEC) strain characteristics.

Strain Name	APEC Isolate	Genome Size (kb)	Serotype	MLST ST	G + C Content (%)	CARD Genes	Virulence Associated Genes	Pro-Phage Regions
B1	AM621	5274.2	O1:H7	95	50.6	52	225	11
B4	AM635	5015.3	O1:H7	95	50.6	44	224	6
B8	AM646	5025.1	O1:H7	95	50.6	44	223	6
B5	AM639	5044.7	O2:H5	355	50.6	46	200	6
B10	AM650	4984.4	O2:H5	355	50.6	43	210	5
B2	AM631	5144.8	O78:H4	117	50.6	46	199	11
B3	AM632	5160.1	O78:H4	117	50.6	50	180	13
B6	AM642	4863.7	O78:H9	23	50.6	51	178	9
B7	AM644	5056.7	O78:H9	23	50.6	51	187	12
B9	AM648	5037.0	O78:H9	23	50.5	50	188	11

ST = Sequence type. CARD: Comprehensive Antibiotic Resistance Database.

**Table 2:** Coliphage characteristics.

Name	Phage Name	Genome Size (kb)	Phage Family	Phage Subfamily	Phage Genus	Accession No.
P10	Phage 60	86.2	*Myoviridae*	*Ounavirinae*	*Felixounavirus*	SRX8360069
P12	Phage 62	87.9	*Myoviridae*	*Ounavirinae*	*Felixounavirus*	SRX8360072
P17	Phage 78	89.9	*Myoviridae*	*Ounavirinae*	*Felixounavirus*	SRX8360088
P18	Phage 79	89.7	*Myoviridae*	*Ounavirinae*	*Felixounavirus*	SRX8360089
P4	Phage 17	45.9	*Drexlerviridae*	*Braunvirinae*	*Guelphvirus*	SRX8360091
P13	Phage 70	44.5	*Drexlerviridae*	*Braunvirinae*	*Guelphvirus*	SRX8360079
P14	Phage 74	46.7	*Drexlerviridae*	*Braunvirinae*	*Guelphvirus*	SRX8360084
P7	Phage 53	50.8	*Drexlerviridae*	*Tempevirinae*	*Hanrivervirus*	SRX8360063
P8	Phage 54	52.6	*Drexlerviridae*	*Tempevirinae*	*Hanrivervirus*	SRX8360064
P16	Phage 77	51.1	*Drexlerviridae*	*Tempevirinae*	*Hanrivervirus*	SRX8360087
P3	Phage 15	169.6	*Myoviridae*	*Tevenvirinae*	*Mosigvirus*	SRX8360082
P5	Phage 18	169.9	*Myoviridae*	*Tevenvirinae*	*Mosigvirus*	SRX8360092
P6	Phage 30	173.4	*Myoviridae*	*Tevenvirinae*	*Mosigvirus*	SRX8360094
P1	Phage 10	169.0	*Myoviridae*	*Tevenvirinae*	*Tequatrovirus*	SRX8360061
P2	Phage 11	171.4	*Myoviridae*	*Tevenvirinae*	*Tequatrovirus*	SRX8360071
P9	Phage 55	170.0	*Myoviridae*	*Tevenvirinae*	*Tequatrovirus*	SRX8360065
P11	Phage 61	109.9	*Demerecviridae*	*Markadamsvirinae*	*Tequintavirus*	SRX8360070
P15	Phage 76	51.9	*Drexlerviridae*	*Tempevirinae*	*Warwickvirus*	SRX8360086

Phage classification according to the current (16 September 2021) International Committee on Taxonomy of Viruses (ICTV) taxonomy.

## Data Availability

Raw reads data for the APEC genome sequences were registered with the NCBI BioProject database and assigned BioProject ID: PRJNA748278.

## Supplementary Materials

The following are available online at https://www.mdpi.com/article/10.3390/microorganisms9122470/s1: Figure S1: Growth dynamics curves for phage-bacterium combinations at all MOIs. Figure S2: Principal component analysis (PCA) of the differences in the OD trajectories/values of the phage–host combinations. Figure S3: Growth dynamics curves for Group 3 subgroups. Table S1: Prophages identified in the APEC genomes. Table S2: Nonmetric multidimensional scaling (NMDS) grouping summary. Table S3: Nonmetric multidimensional scaling (NMDS) Group 3 subgrouping summary.
