# Supplementary material for: Classification of In Vitro Phage–Host Population Growth Dynamics
Source: Microorganisms. 2021 Nov 30;9(12):2470. doi: 10.3390/microorganisms9122470 (PMC8708399; doi:10.3390/microorganisms9122470)
Supplement: Supplementary file 1 [file microorganisms-09-02470-s001.zip › microorganisms-1459555-supplementary.pdf]

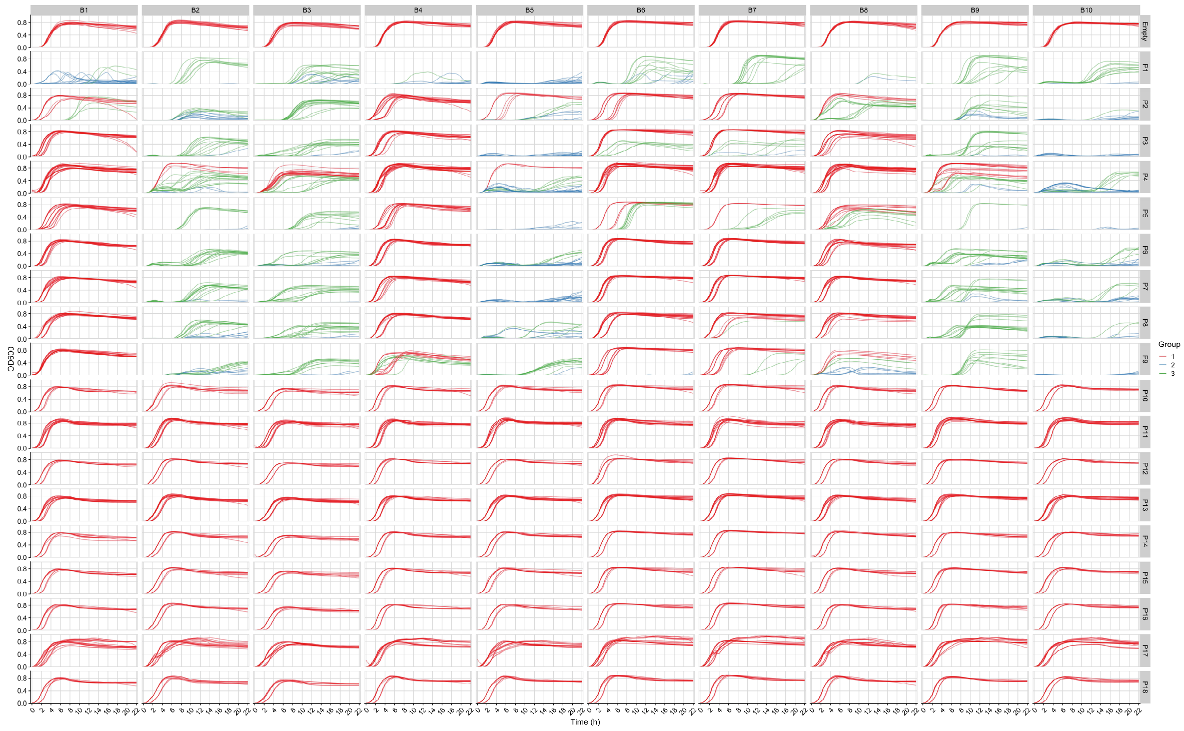

Figure S1. Growth dynamics curves for phage-bacterium combinations at all MOIs.

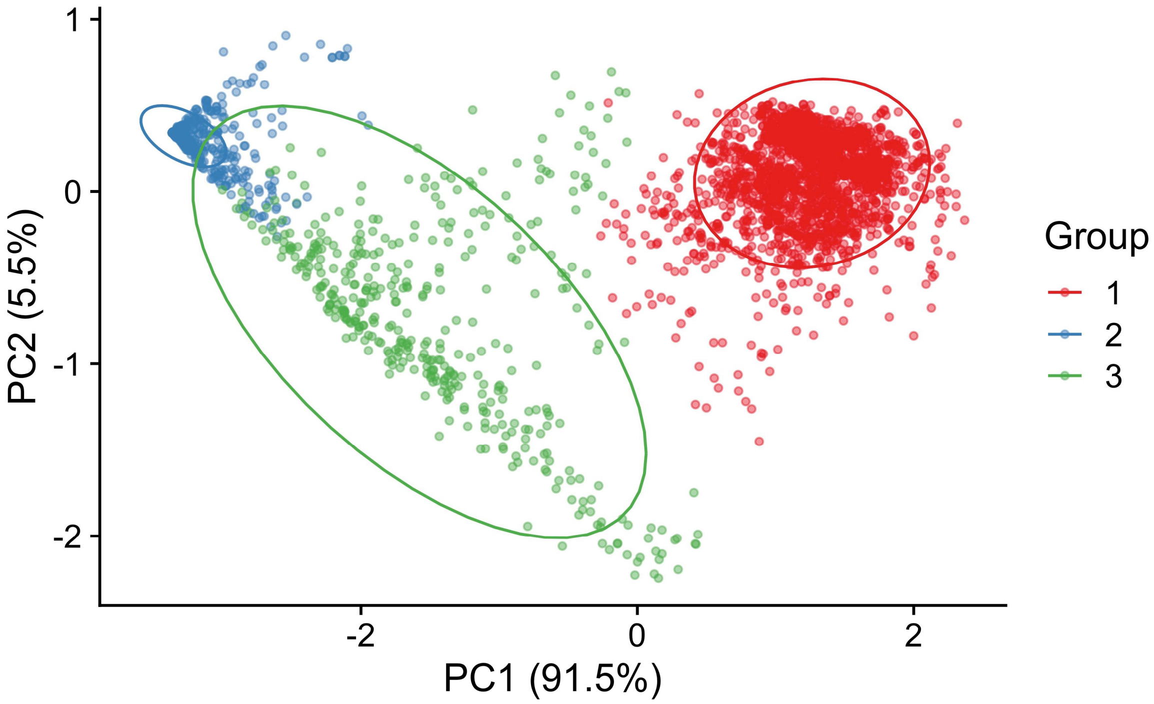

Figure S2. Principal component analysis (PCA) of the differences in the OD trajectories/values of the phage–host combinations.

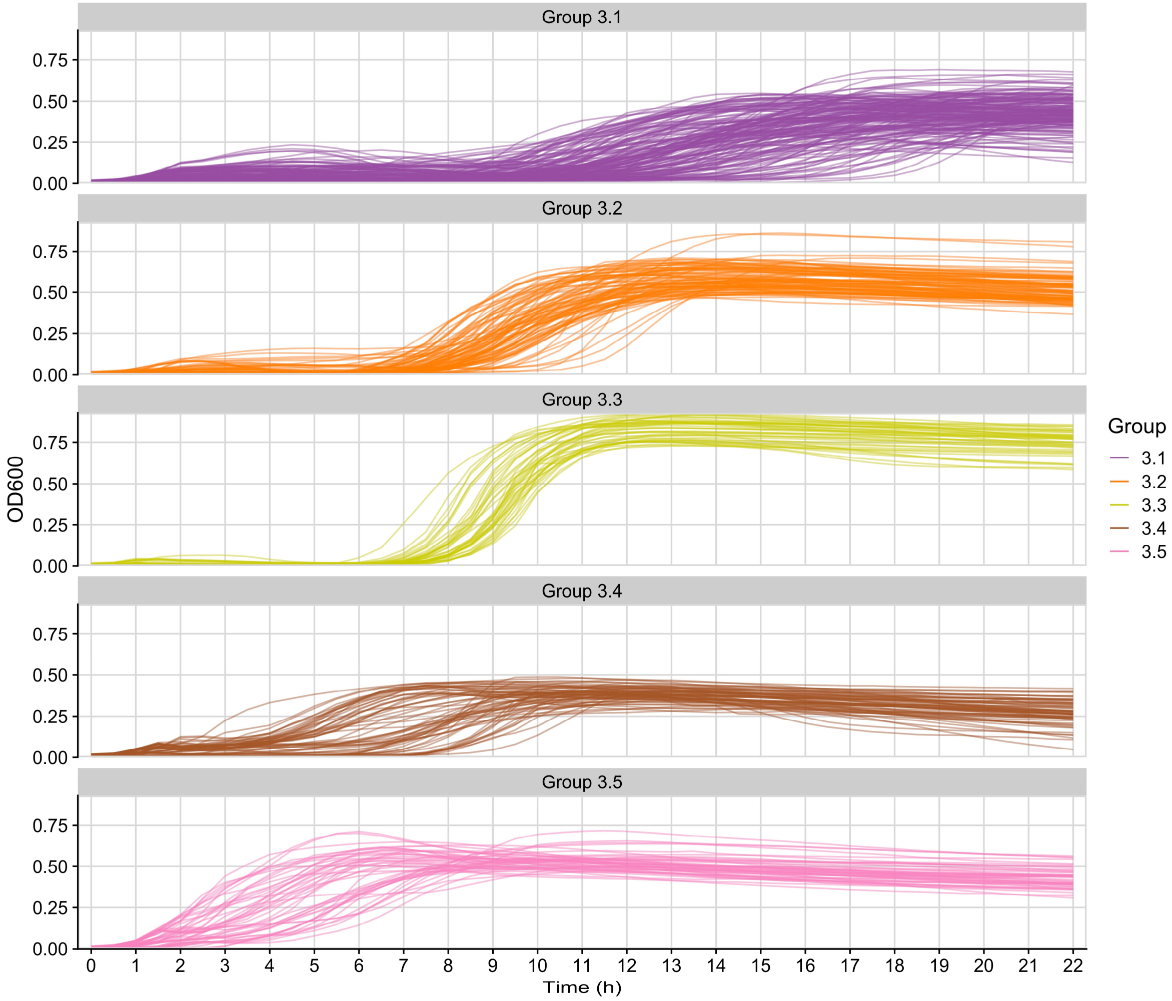

Figure S3. Growth dynamics curves for Group 3 subgroups.
